# An empirical evaluation of The Resilience Shield model

**DOI:** 10.1186/s40359-022-00891-9

**Published:** 2022-07-23

**Authors:** Lies Notebaert, Hannah Abdul Razak, Stijn Masschelein

**Affiliations:** 1 Centre for the Advancement of Research on Emotion, School of Psychological Science, University of Western Australia, M304, 35 Stirling Highway, Crawley, WA 6009 Australia; 2 Accounting and Finance Department, Business School, University of Western Australia, Crawley, Australia

**Keywords:** Resilience, The Resilience Shield, Mind, Body, Social, Professional

## Abstract

**Background:**

Resilience refers to the process through which individuals deal with the adversity they experience. Previous research has shown there are multiple factors that contribute to individuals’ resilience, leading to increasing interest in the development of multidimensional resilience models. Once such recently proposed model is The Resilience Shield, which clusters groups of protective factors into different shield layers. The stronger these layers, the better the protection against adversity (Pronk et al. in The Resilience Shield, Pan Macmillan Australia, 2021). While this model was based in part on existing literature, no empirical evaluation has occurred to date. The aim of this study was therefore to evaluate the model fit for each of the modifiable shield layers and the overall model, and to examine whether each of the constructs included contributes to observed resilience scores.

**Methods:**

Participants completed a series of questionnaires via The Resilience Shield website assessing constructs relevant to each resilience shield layer. Data from 3337 participants was analysed using Structural Equation Modelling and regression analyses.

**Results:**

The results showed acceptable fit of the measurement model for the Social, Mind, and Professional Layers, but poor fit for the Body Layer. There was also good fit for the overall model. In addition, all but one of the constructs included in The Resilience Shield survey explained independent variance in either dispositional resilience scores, or dispositional vulnerability scores.

**Conclusion:**

These results broadly support the multidimensional structure proposed by The Resilience Shield model and suggest that (at least in the population in which it was tested) this may be an acceptable model to index individuals’ performance on a range of indicators that contribute to resilience.

## Background

Resilience refers to “the process of adapting well in the face of adversity, trauma, tragedy, threats or even significant sources of stress” [2, paragraph 4]. Greater resilience has a positive impact on individuals’ functioning in a range of domains, including psychological, physiological and occupational domains. Positive outcomes associated with greater resilience include longevity, lower rates of mental health disorders, greater well-being, better work performance and increased life satisfaction [47, 52, 98]. Psychologically, enhanced resilience has been shown to be a significant protective factor against psychopathologies [4] as greater resilience is associated with lower rates of emotional vulnerability and psychological distress [38] as well as a reduced likelihood of PTSD symptomology following an adverse event [73, 97]. Greater resilience is also associated with better physical health [67, 102], and has shown to maximize benefits from treatment interventions for illnesses such as coronary heart disease and diabetes [18, 106]. In a workplace setting, more resilient individuals are less likely to experience frequent stress and suffer from burnout [64, 95]. Hence, the study of resilience can provide invaluable implications in practical and theoretical fields of mental and physical health, as well as in organisations.

Several approaches have been taken to measure resilience in individuals [104]. One approach is to ask people to report on their level of resilience directly, for example by asking individuals to report how well they bounce back from adversity. This is the approach employed for example in the Brief Resilience Scale [91]. A downside of this approach is that it might be difficult for individuals to have insight in their own resilience, as this is a complex, abstract concept. Research has suggested that self-reports of such constructs are less reliable than self-reports on for example patterns of behaviour, as people have limited introspective access to abstract psychological concepts [59, 69].

Alternatively, the processes that are thought to contribute to more resilient outcomes can be measured. The benefit of this approach is that individuals find it easier to report on more specific and more salient behaviours, thoughts, and goals, and such reports tend to be more accurate [69]. An example of this approach to measuring resilience is the Connor-Davison Resilience Scale, where the 25 items cover a range of hypothesized predictors of resilience (CD-RISC) [26]. The downside of the latter approach however, is that using items that reflect hypothesized contributing factors compromises researchers' ability to test hypotheses regarding the factors that may underpin individual differences in resilience. For such research, it is imperative to use a measure that is not already confounded by the inclusion of potential predictors. For example, the CD-RISC includes items reflecting the use of humour and having a strong sense of purpose. Any research examining the contribution of humour and purpose to resilience using the CD-RISC as the outcome measure is therefore likely to over-estimate the contribution of these factors.

Different research areas have focused on understanding the mechanisms that contribute to enhanced resilience. For example, cognitive psychologists have sought to identify the individual cognitive processes that enhance resilience (e.g. [77, 86]). Others have focused on affective mechanisms (e.g. [8, 10]), developmental perspectives (e.g. [63]), skills and personality attributes (e.g. [26]), biological factors [29], or physical health characteristics (e.g. [88, 89]). While such research provides valuable insight into the determinants of resilience, increasingly researchers and clinicians are emphasizing the need for comprehensive models that approach resilience from multiple domains, as factors in each individual domains will inevitably only explain a small portion of the resilience puzzle [43, 92].

One such multi-dimensional model recently proposed is The Resilience Shield [80]. The Resilience Shield is a theoretical model of resilience developed by Dr Dan Pronk, Ben Pronk DSC and Tim Curtis, all of whom are Australian Army veterans who served for extensive periods within the Special Air Service Regiment. Their interest in the concept of resilience originates from their observations of the chronic and acute reactions of themselves and their colleagues following exposure to periods of stress, including significant exposure to combat in theatres such as Afghanistan, Iraq, Timor Leste and Sierra Leone. They observed markedly different reactions to these stressors amongst their peers, an ostensibly homogenous cohort of individuals who had all been screened for above average mental and physical robustness through the unit’s selection process. This initial observation served as a catalyst for a literature review on the topic of resilience which led to the conclusion that resilience was *dynamic* (in that it can change within an individual as a result of time, circumstance and the domain specificity of the stressor), *modifiable* (it can be increased through deliberate and targeted intervention) and *multifactorial* (there are multiple constituent elements that contribute to enhanced resilience).

Based on their literature review, the authors observed that the key constituent elements of resilience identified in existing resilience research could be grouped along thematic lines. Moreover, they identified that there did not appear to be a holistic model of resilience that looked at how these thematic groups interacted with each other. Based on this, they developed the Resilience Shield model. The Resilience Shield refers to these thematic groups of resilience factors as ‘layers’.

**Fig. 1:**
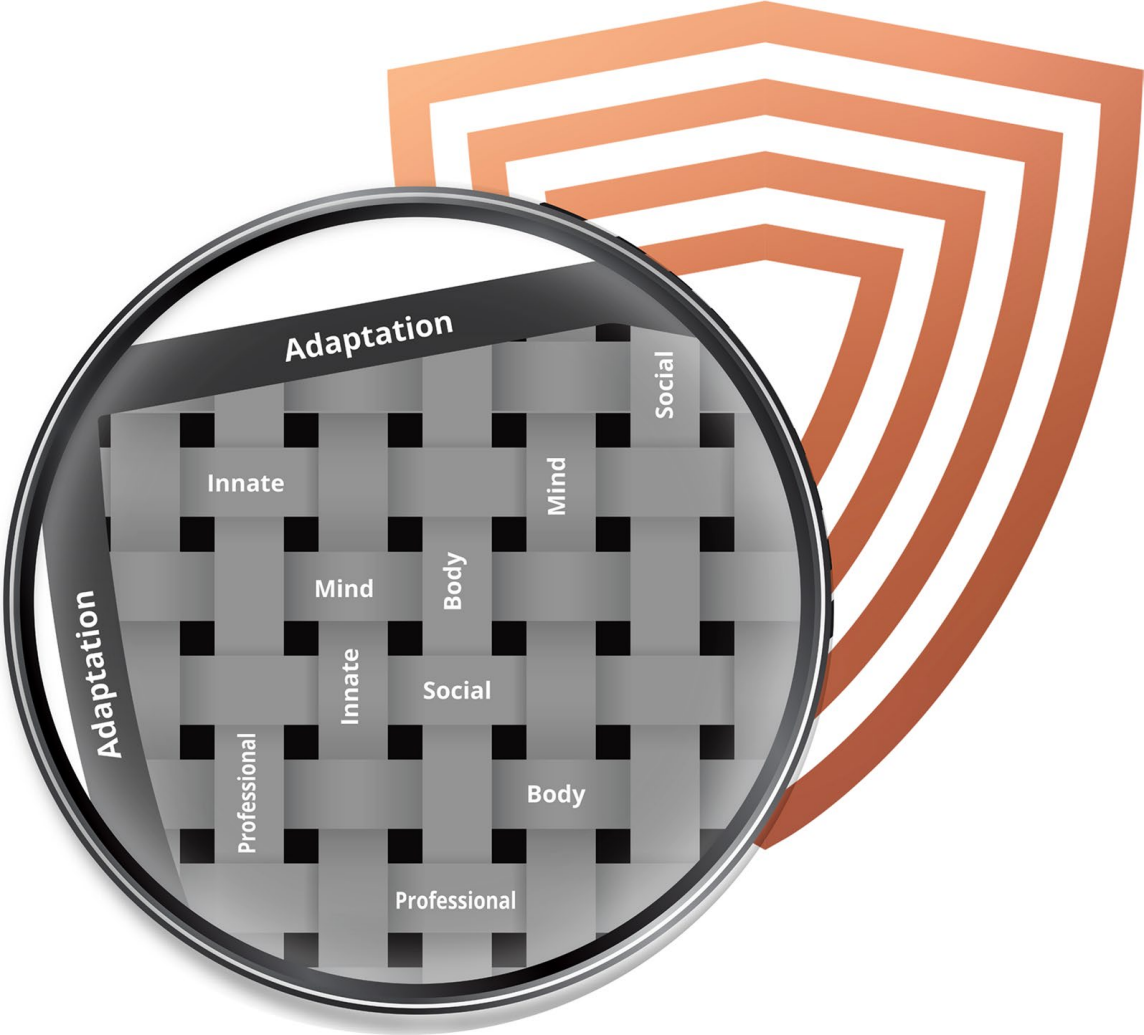
The weave-like pattern through which the different layers of the Resilience Shield are connected

The Innate Layer consists of genetic and epigenetic factors; The Mind Layer consists of psychological and spiritual factors; The Body Layer consists of physiological factors; The Social Layer consists of factors related to support from others; The Professional Layer consists of vocational factors. Finally, the Adaptation Layer is an overarching layer, which recognises that domain-specific resilience developed in the previous layers has transferability to novel challenges. The Resilience Shield model further proposes that these layers do not exist in isolation, but rather interact with one another in a weave-like fashion, as depicted in Fig. 1. Finally, the model was designed to be universally applicable, providing relevance and insight to individuals across a wide range of demographic and geographic characteristics. The Resilience Shield model was codified in a book, *The Resilience Shield*, published by Pan MacMillan in August 2021 [80].

The Resilience Shield model was developed by amalgamating existing empirical findings. Indeed, there is empirical support for an association between the constructs captured in each of the Layers and variation in resilience. Relevant to the Mind Layer, there is an abundance of evidence showing that cognitive processes contribute to resilience. One such construct is *cognitive flexibility*, defined as the ability to adaptively switch cognitive sets to better suit the environment [32]. Multiple studies have found a significant positive correlation between cognitive flexibility and resilience [74] showing for example that more resilient individuals can flexibly apply different coping strategies depending on the circumstances, rather than being set in implementing only one strategy [93]. The construct of *mindfulness*, defined as the ability to intentionally focus attention on the present moment non-judgementally [46], has also been shown to be a significant predictor of resilience [44, 48, 72]. Practicing the skill of mindfulness brings awareness to the transient nature of negative thoughts, emotions and bodily sensations, which in turn allows for an individual to respond objectively rather than reacting reflexively [46, 60]. Several studies have also shown the construct of *gratitude*, defined as the quality of being thankful and readiness to show appreciation [82], to be a significant contributor to resilience [36, 62]. Lastly, the construct *locus of control*, referring to an individual’s beliefs on whether outcomes occur as a direct result of their own actions or due to external factors such as chance, has also been suggested to contribute to resilience [35]. Specifically, multiple studies have demonstrated that individuals with an internal locus of control, the belief that outcomes are a direct result of their own actions, show higher levels of resilience compared to those with an external locus of control [17, 35, 73].

It has also long been known that physical factors make an important contribution to resilience. Research has shown for example that more resilient people tend to exercise more [20] and have a healthier diet [70]. Moreover, a sufficient amount of quality sleep appears to be an important predictor of resilience. Studies have shown that good sleepers are more resilient than poor sleepers, with these individuals showing higher sleep efficiency, less light sleep, more slow-wave sleep and less awakenings throughout their sleep [55]. Further, another study investigating the association between sleep, resilience and psychological distress in veterans found that whilst poor sleep was associated with greater psychological distress, resilience was found to have significant buffering effects on this relationship, suggesting that greater resilience may also protect individuals from the negative impacts of poor sleep [42]. A possible reason for this could be due to sleep and resilience sharing similar neuronal networks and brain hubs [99]. These research findings led to the inclusion of a Body Layer in the Resilience Shield model.

The inclusion of a Social layer was based on the literature showing that social support is a key determinant of resilience, independent of gender, age, education and income level, and ethnicity [9]. Social support can be described as “the provision of a social network’s psychological and material resources intended to benefit an individual’s capacity to cope with stress” [90]. One study conducted found that individuals' PTSD symptoms were less likely to lead to suicidal behaviour if they had high perceived social support, a finding which highlights the contribution of social support to developing stronger resilience [76]. Additionally, poorer social support has shown to be associated with increases in morbidity and mortality in a range of medical illnesses, whereas higher levels of social support have been shown to buffer or protect against the full impact of these illnesses [6, 75].

Finally, the inclusion of the Professional layer was derived from literature in the domain of work psychology that has shown how work-related attributes can also contribute to better resilience. For example, the job demands/resources model proposes that health-protecting factors known as job resources, are what keep people healthy at work in the face of high job demand. Job demands are the aspects of a job that require sustained physiological and psychological efforts, and are associated with negative costs [27, 34]. Constructs which contribute towards job resources include occupational self-efficacy and job autonomy, whilst workload contributes towards job demands [34]. Job autonomy, defined as the degree to which employees have control and discretion over how to carry out duties [83] and occupational self-efficacy, defined as employees’ belief in their own competence to successfully and effectively perform across different situations and tasks in a job [87], have both shown to be significantly correlated with resilience [34]. Whilst workload is associated with poorer outcomes [34] studies have shown that greater resilience is associated with lower levels of heavy workload-induced distress [50]. Lastly, it is theorised that individuals’ sense of purpose in the workplace also contributes to resilience, as studies have shown that individuals who report having a greater sense of purpose demonstrate quicker and more efficient emotional recovery following negative events [85].

While The Resilience Shield model is based on existing empirical findings, it remains to be seen whether the model itself is empirically supported. This is critical if the model is to serve as the basis for assessing individuals’ resilience level to identify areas where improvements could be made, and/or as the basis for evaluating the success of resilience building interventions or programs. As such, the aim of the current study was to investigate the validity of the model by i. examining whether each of the constructs identified in each of the layers contribute a “Layer” latent construct, ii. examining whether each of the layers contribute to a higher order latent construct (“resilience”), and iii. examining whether each of the constructs constituting each layer contributes to observed resilience scores. As the primary value of this model lies in the contribution to improving resilience, only the modifiable layers of the resilience Shield were evaluated (i.e. Mind, Body, Social, and Professional, excluding the Innate and Adaptation Layers).

To do so, data collected through the Resilience Shield survey hosted on the developers’ website (resilienceshield.com) was analyzed using Structural Equation Modeling and regression analyses. Given the large number of constructs The Resilience Shield survey aimed to address, the number of items to index each construct was minimised to prevent drop-out and survey fatigue. As such, the instrument items incorporated into this Resilience Shield survey were guided by the results from two pilot studies, of which relevant details are also reported.

Overall resilience was measured using the Hardiness Scale [58], which circumvents the two issues relating to the measurement of resilience described above. The scale does not ask people to reflect on their general resilience level, circumventing the problems associated with people’s limited introspection. Instead, the scale asks about specific thoughts and feelings that are reflective of individuals that are more resistant to stress and adversity, and thoughts and feeling that are characteristic of individuals who are highly sensitive to stress and adversity (an approach akin to measures of personality). As the scale does not include items reflecting any of the constructs included in the Resilience Shield model layers, this bypasses the issue of the measure of resilience incorporating possible predictors identified in the Resilience Shield model. Another major benefit of the Hardiness Scale is that it assesses both Resilience factors and Vulnerability factors. Resilience factors are assets, resources, characteristics and skills which may reduce the risk of negative outcomes after or during an acute or chronic stressor. Vulnerability factors on the other hand are factors which increase the probability of negative outcomes when faced with adversity [58].

## Methods

### Methods Pilot Study 1

Pilot Study 1 was designed to assess the reliability of the instruments designed to capture constructs in the Mind layer and Professional layer, and critically to determine the subset of instrument items to be used in the main study.

Participants were recruited through Amazon Mechanical Turk among US-based workers with > 5000 completed Human Intelligence Tasks (HITs), and an approval rate of > 98%. Four-hundred and fifty participants were recruited. Their average age was 43.81, SD = 12.55; 50.9% identified as male, 48.7% as female, one participant identified as non-binary, and one preferred not to disclose gender information. Most participants (84.7%) were White American, European American, or Middle Eastern American, 6.7% were Black or African American, 6.2% were American Indian or Alaska Native, five participants were multi-racial, and another six chose the ‘Other’ response option. Most participants were either married (46%) or never married (38.9%), some were divorced (12%) or separated (1.1%), while a small number were widowed (2%). Most participants had an undergraduate (49.6%) or postgraduate (16.2%) degree, others obtained a high school degree (18.7%), doctorate (5.1%), diploma (4.4%), or trade degree (4.2%). A smaller number did not complete high school (1.3%) or preferred not to disclose educational qualifications (0.4%).

After providing written informed consent, participants were administered the instruments relating to the Mind layer and Professional layer described below. The average time to complete the battery of questionnaires was 13.76 min. Participants were reimbursed 2USD.

### Methods Pilot Study 2

Data from Pilot Study 2 were used to assess the reliability of the instruments designed to capture constructs in the Social layer, and critically to determine the subset of instrument items to be used in the main study.

Participants were recruited through The Resilience Shield Website and social media platforms. The data were downloaded on 19 February 2021, at which point 968 participants had completed the survey, with a mean age of 39.47 (SD = 11.35). The majority of participants was female (58.26%), with 41.43% identifying as male, and 3 participants as identifying as non-binary. Most participants were living in Australia (90.70%), with a minority residing in the UK (2.38%), the US (2.07%), or New Zealand (0.83%). Almost half of participants were married (47.35%), with other reporting their relationship status as engaged (5.17%), living with a partner (14.77), or in a relationship (6.40%). Others were single (20.76%), divorced (1.14%), or separated (1.76%). A small number of participants ticked the ‘Other’ option (0.72%). Most participants had an undergraduate (29.44%) or postgraduate (36.36%) degree, others obtained a high school degree (8.37%), diploma (11.36%), trade degree (4.03%), or doctorate (8.3%). A smaller number did not complete high school (1.76%) or preferred not to disclose educational qualifications (0.31%).

After providing written informed consent, participants were administered a battery of questionnaires including the instrument relating to the Social layer described below. The battery of questionnaires took about 25 min to complete. Participants were not reimbursed.

### Methods main study

#### Participants

Participants were recruited through The Resilience Shield book, social media platforms, and direct author engagements, including corporate consulting engagements and keynote presentations. While data collection is ongoing, data for this study were downloaded on November 8, 2021, at which point in time 3990 participants had started the survey and 3564 had completed it. In the final sample, by far the majority of participants resided in Australia (86.4%), with a small percentage residing in the United States (2.5%), New Zealand (1.8%), the United Kingdom (1.5%), and Canada (1%). The majority of participants identified as female (62.1%), with 37.3% male participants and 0.6% who identified as non-binary or preferred not to disclose gender. Mean age was 39.5, SD = 12.07, range 16–84.

#### Questionnaires

##### Demographic questions

The following demographic information was collected: Gender, Date of birth, Employment sector, Country of residence, Highest educational qualification, Annual gross household income bracket, and Relationship status.

##### Resilience

Resilience was measured using an abbreviated version of the 18-item Dual-Process Hierarchical Scale of Hardiness developed by Lo Bue [58]. This questionnaire measures both the process of dispositional resilience (which provides strength and resources when confronted with adversity) and the process of dispositional vulnerability (which increases sensitivity to stressors). Dispositional Resilience is indexed across three three-item subscales: Commitment (e.g. ‘I really look forward to the tasks I have to do each day’), Control (e.g. ‘I feel confident I can handle just about any challenge’), and Challenge (e.g. ‘I'm always seeking for new challenges to overcome’). Likewise, Dispositional Vulnerability is indexed across three three-item subscales: Alienation (e.g. ‘Sometimes, life is meaningless to me’), Powerlessness (e.g. ‘No matter how hard I try, my efforts usually accomplish nothing’), and Rigidity (e.g. ‘I don't like to make changes in my regular activities’). Respondents are asked to indicate how true each statement is for them on a five-point Likert scale (1 = Not at all true, 2 = Slightly true, 3 = Moderately true, 4 = Very true, 5 = Completely true). Higher scores indicate that respondents see themselves more as having the specific thoughts and feelings that are reflective of individuals that are more resistant to stress and adversity (dispositional resilience), and less as having the specific thoughts and feeling that are characteristic of individuals who are highly sensitive to stress and adversity (dispositional vulnerability).

SEM analyses on data from Pilot Study 1 which administered the full 18-item version of this instrument provided support for a correlated dual process model with the latent constructs Dispositional Resilience and Dispositional Vulnerability indexed by their respective sub-constructs as measured by the three items relevant to these scales, Chi squared = 294.402, *p* < 0.001, CFI = 0.997, RMSEA = 0.055. Cronbach’s alpha for each of the subscales was high: Commitment = 0.906, Control = 0.840, Challenge = 0.838, Alienation = 0.879, Powerlessness = 0.888, Rigidity = 0.899; as was the reliability of the Dispositional Resilience total scores (Cronbach’s alpha = 0.922) and the Dispositional Vulnerability total scores (Cronbach’s alpha = 0.864).

In the current study, the highest loading item on each subscale (the example items described above) for each of the two processes was retained, with their scores averaged to create a Dispositional Resilience Score (Cronbach’s alpha = 0.733) and a Dispositional Vulnerability Score (Cronbach’s alpha = 0.559). Higher scores therefore reflect greater resilience and vulnerability, respectively.

##### Mind layer instruments

*Mindfulness*. Mindfulness was measured using an abbreviated version of the 15-item Mindful Attention Awareness Scale [12]. This scale asked respondents to report how frequently they experience a range of mindful states in everyday life, on a six-point Likert scale ranging from ‘Almost never’ to ‘Almost always’. The scale reports strong psychometric properties and has been validated with college, community, and cancer patient samples [12].

Data from Pilot Study 1 which administered the full 15-item version of this instrument showed high internal consistency, Cronbach’s alpha = 0.933. A SEM showed that all items loaded onto a common latent factor, χ2(90) = 205.25, *p* < 0.001, CFI = 0.99, RMSEA = 0.06. The two items with the highest R^2^ were retained in the current study (‘It seems I am "running on automatic," without much awareness of what I'm doing’, and ‘I rush through activities without being really attentive to them’). Scores on these items were reverse coded and averaged to calculate a Mindfulness Score, with higher scores indicating a higher frequency of experiencing mindful states.

*Gratitude*. Gratitude was measured using an abbreviated version of the Gratitude Questionnaire [65]. In this six-item survey, respondents indicate their agreement with various statements about experiencing gratitude in everyday life, on a seven-point Likert scale ranging from ‘Strongly disagree’ to ‘Strongly agree’.

The scale reports good psychometric properties [65]. In Pilot Study 1 which administered the full six-item version of this instrument, internal consistency was likewise high with Cronbach’s alpha = 0.896. A SEM showed that all items loaded onto a common latent factor, χ2(9) = 53.86, *p* < 0.001, CFI = 0.99, RMSEA = 0.11. The two items with the highest R^2^ were retained in the current study (‘I have so much in life to be thankful for’, and ‘If I had to list everything that I felt grateful for, it would be a very long list.’). Scores on these two items were averaged to produce a Gratitude Score, with higher scores indicating a greater disposition to experience gratitude.

*Locus of Control*. Locus of Control was measured using an abbreviated and modified version of Rotter’s Internal External Locus of Control Scale [39, 81]. This original scale is a 29-item force choice paradigm where respondents must choose between an internal or external interpretation of a situation (e.g. ‘Becoming a success is a matter of hard work, luck has little or nothing to do with it.’ Versus ‘Getting a good job depends mainly on being in the right place at the right time.’, respectively). The number of external relative to internal interpretations respondents endorse is taken to reflect respondents’ locus of control.

Rotter’s Internal External Locus of Control Scale has acceptable psychometric properties, including validity and reliability [54, 96, 108]. Data from Pilot Study 1 which administered the full 29-item version of this instrument showed high internal consistency, Cronbach’s alpha = 0.847. A SEM showed that all items loaded onto a common latent factor, χ2(209) = 1156.59, *p* < 0.001, CFI = 0.90, RMSEA = 0.10. For the current study, the two highest loading items were retained (the example item above, and ‘Getting people to do the right thing depends upon ability. Luck has little or nothing to do with it.’ versus ‘Who gets to be the boss often depends on who was lucky enough to be in the right place first.’). As the scale was reduced to two items, the response format was changed to allow for a better and more fine-grained assessment of locus of control as compared to a forced-choice measure [51]. Specifically, both interpretations of each item were presented, and respondents were asked to indicate their agreement with each of a five-point Likert scale ranging from ‘Strongly disagree’ to ‘Strongly agree’. After reverse coding the external interpretations, responses on the resulting four item-scale had low internal consistency (Cronbach’s alpha = 0.546). These scores were averaged to produce a Locus of Control Score, with higher scores indicating a more internal locus of control.

*Cognitive flexibility*. Cognitive flexibility was assessed with an abbreviated version of the ‘Alternatives’ subscale of the Cognitive Flexibility Inventory [32]. This scale assesses the ability to generate multiple alternative solutions to difficult situations, by asking respondents to indicate their agreement with 13 statements (e.g. ‘I consider multiple options before responding to difficult situations’) on a seven point Likert scale ranging from ‘Strongly disagree’ to ‘Strongly agree’.

The Cognitive Flexibility Inventory has high internal consistency, a reliable factor structure, high test–retest reliability, and good convergent and concurrent construct validity [32]. Data from Pilot Study 1 which administered all 13 items of this instrument subscale also showed high internal consistency, Cronbach’s alpha = 0.910. A SEM showed that all items loaded onto a common latent factor, χ2(65) = 535.54, *p* < 0.001, CFI = 0.99, RMSEA = 0.13. For the current study, the two items with the highest R^2^ were retained (the example item above, and ‘I often look at a situation from different viewpoints.’). Scores on these two items were averaged to produce a Cognitive Flexibility Score, with higher scores indicating greater cognitive flexibility.

##### Body layer instruments

*Sleep*. Sleep was measured using the overall sleep quality question in the Pittsburgh Sleep Quality Index [16]. This question asks respondents to rate their sleep quality in the past month on a four point Likert scale ranging from ‘Very bad’ to ‘Very good’. Higher scores therefore indicate better sleep quality.

*Nutrition*. Nutrition was assessed using a custom developed instrument based on Australian government dietary guidelines, asking respondents to indicate how many servings of vegetables they eat each day (< 4, 4–7, > 7), how many servings of fruit (0–1, 2, ≥ 3), how much water they drink each day (< 2 l, 2-3 l, > 3 l), how many times a week they overeat (never, 1–3 times, > 3 times), and how many times a week they eat takeaway food (never, 1–3 times, > 3 times). In addition, respondents were asked to indicate their agreement with the statement that their diet is well balanced, including food from all food groups most days, on a five-point Likert scale ranging from ‘Strongly disagree’ to ‘Strongly agree’. After rescaling responses to the latter question to a three-point scale and reverse scoring the question on the overeating and take-aways items, item scores were averaged to produce an overall Nutrition Score, with higher scores indicating better nutrition. As the items in this instrument were not all designed to measure the same construct, Cronbach’s alpha was not calculated. Scores on the balance question shows small to moderate correlations with the other questions, ranging from 0.134 (water consumption) to 0.351 (vegetable consumption), demonstrating the construct validity of these questions.

*Exercise*. Exercise was measured using an abbreviated version of the World Health Organisation’s Global Physical Activity Questionnaire [24]. This instrument has been shown to be a reliable and valid measure of moderate and vigorous activity [14, 101]. In the current study, respondents were asked to indicate how many days a week they engage in vigorous activity, and on these days, how much time on average is spent doing vigorous activity. The same two questions were asked in relation to moderate activity. For each activity type (rigorous and moderate), the number of days engaged in it was multiplied by the average time spent in it. In accordance with scoring guidelines [103], these multiplied scores were then combined, weighting the rigorous score double to create an overall Exercise Score. Higher scores indicate greater engagement in vigorous and moderate activity.

##### Social layer instruments

*Social support*. Social support was measured using an abbreviated version of the Multi-Dimensional Scale of Perceived Social Support (MSPSS; [109]. This 12-item instrument asks respondents to indicate how much they agree with various statements describing the ability to gain support from friends (e.g. “I can count on my friends when things go wrong”), family (e.g. “I get the emotional help and support I need from my family”), and a significant other (e.g. “There is a special person with whom I can share my joys and sorrows”). One of the most commonly used measures of social support, the MSPSS has strong factorial validity, good internal and test–retest reliability, and moderate construct validity [109].

Data from pilot study 2 showed that each subscale had high internal consistency: Cronbach’s alpha Friends subscale = 0.835, Cronbach’s alpha Family subscale = 0.913, Cronbach’s alpha Significant Other subscale = 0.955. A SEM with each subscale presented as a latent factor loading onto a higher order latent factor showed an acceptable fit, χ2(51) = 158.37, *p* < 0.001, CFI = 0.99, RMSEA = 0.05. The two items with the highest R^2^ for each subscale were retained (the example items above, and “I have friends with whom I can share my joys and sorrows”, “I can talk about my problems with my family”, and “There is a special person who is around when I am in need”).

##### Professional layer instruments

*Occupational self-efficacy*. Occupational self-efficacy was assessed using an abbreviated version of the short form of the occupational self-efficacy scale developed by Schyns and von Collani [87]. In this eight-item instrument, respondents are asked to indicate to what extent they believe various items describing perceived competence at work are true, on a six-point scaling ranging from ‘Not at all true’ to ‘Completely true’. An example item is ‘Thanks to my resourcefulness, I know how to handle unforeseen situations in my job’. This is a reliable, one-dimensional instrument with acceptable construct and criterion validity [87].

Data from Pilot Study 1 which administered all eight items also showed high internal consistency (Cronbach’s alpha = 0.950). A SEM showed that all items loaded onto a common latent factor, χ2(14) = 65.62, *p* < 0.001, CFI = 0.99, RMSEA = 0.09. The two items with the highest R^2^ were retained (the example item above and ‘No matter what comes my way in my job, I’m usually able to handle it’). Scores on these two items were averaged to produce an Occupational Self-Efficacy Score, with higher scores indicating greater occupational self-efficacy.

*Sense of Purpose at Work*. Sense of purpose within the workplace was assessing using the Meaning subscale of the PERMA-profiler questionnaire [15]. This instrument demonstrates acceptable model fit, internal and cross-time consistency, and evidence for content, convergent, and divergent validity [15]. An example item is ‘To what extent do you feel that what you do in your life is valuable and worthwhile?’. Respondents are asked to indicate how much each statement applies to them, on an 11-point scale ranging from ‘Not at all’ to ‘Completely’.

In Pilot Study 1, the three items that constitute the Meaning subscale were rephrased to relate to ‘work’ rather than life (i.e. ‘To what extent do you feel that what you do at work is valuable and worthwhile?’). These items showed high internal consistency, with Cronbach’s alpha = 0.956. As with three items a SEM was saturated, retention of items was based on the item-rest correlation of Cronbach’s alpha reliability statistics. The two items with the highest item-rest correlation were retained (the example item above and ‘To what extent is your work purposeful and meaningful?’). Scores on these two items were averaged to produce a Work Purpose Score, with higher scores indicating greater sense of purpose at work.

*Job Autonomy*. Job autonomy was measured using a revised version of the autonomy subscale of the Job Diagnostic Survey [37], as reported by Gardner [34]. Respondents are asked to indicate the accuracy of each statement as it applies to them using a seven-point scale ranging from ‘Very inaccurate’ to ‘Very accurate’. The two items are ‘My job gives me considerable opportunity for independence’ and ‘My job gives me the chance to use my personal initiative and judgement in carrying out the work’. The construct validity of this measure is evident through its correlations with job-related outcome measures such as job satisfaction, organisational commitment, and organisation-based self-esteem [34]. Scores on these two items were averaged to produce a Job Autonomy Score, with higher scores reflecting greater job autonomy.

*Workload*. Workload was assessed using the Quantitative Workload Inventory [94]. This five-item instrument asks respondents to indicate how often various instances of high workload occur (e.g. ‘How often does your job require you to work very hard?’), on a scale with five response options: Less than once per month, Once or twice per month, Once or twice per week, Once or twice per day, and Several time per day. The scale shows good internal consistency and acceptable construct validity [94].

Data from Pilot Study 1 which administered all five items of this instrument subscale showed high internal consistency, Cronbach’s alpha = 0.895. A SEM showed that all items loaded onto a common latent factor, χ2(5) = 36.44, *p* < 0.001, CFI = 0.99, RMSEA = 0.12. The two items with the highest R^2^ were retained (the example item above, and ‘How often is there a great deal to be done?’). Scores on these two items were averaged to produce a Workload Score, with higher scores indicating higher workload.

### Procedure

Participants first provided written informed consent, and then completed the instruments in the following order: resilience, sleep, exercise, nutrition, mindfulness, gratitude, cognitive flexibility, locus of control, social support, occupational self-efficacy, and sense of purpose at work. Demographic questions were peppered throughout the survey. This study and the pilot studies were approved by the Human Research Ethics office at the University of Western Australia.

## Results

### Data cleaning

Respondents’ data were removed if they did not finish the survey (N = 431), or if they recorded an age below 16 (as per the approved ethics protocol, N = 39), or if they specified they were currently retired or unemployed (N = 206). The latter criterion was applied to restrict the sample to people who were active in the workforce, as The Resilience Shield Model includes a Professional Layer which represents the way in which work contributes to resilience.

Data from three participants who selected a year of birth among the first few response options (suggesting they were born in the early 1900s) were removed. After standardizing all variables, any participant who had a standardized instrument score (see list in Table 2) of < − 3.5 or > 3.5 was removed (N = 110). This approach eliminates the top 0.05% and bottom 0.05% of normally distributed scores as outliers [11, 25, 79]. The final sample consisted of 3337 participants.

### Sample characteristics

Over half the sample was either engaged (4.1%), married (50.3%) or living with a partner (15.6%). A smaller proportion reported being single (17.3%) or being in a relationship but not living together (7.4%). A smaller group of participants was divorced (2.6%) or separated (2.0%). A small number of participants was widowed (0.6%).

As can be seen in Table 1, a large percentage of participants worked either in healthcare or social assistance (18.4%) or in the military (14.7%). The two other large employee group were people in professional, scientific or technical services (10.4%) and first responders (7.9%). Education levels were generally high, with over half the sample obtaining an undergraduate degree (27.2%), postgraduate degree (27%), or doctorate (12.7%).

**Table 1:** Frequency tables of employer category and education level

Employer	N	%	Education	N	%
Forestry, fishing or agriculture	53	1.64	No response	3	0.09
Real estate or rental and leasing	26	0.81	Rather not say	19	0.59
Resources, including Mining and Oil & Gas	134	4.15	Did not complete high school	73	2.26
Professional, scientific or technical services	336	10.41	High school graduate	386	11.96
Utilities	23	0.71	Undergraduate degree	876	27.15
Management of companies or enterprises	173	5.36	Trade	165	5.11
Construction	122	3.78	Diploma	423	13.11
Admin, support, waste management or remediation services	86	2.67	Postgraduate degree	871	26.99
Manufacturing	86	2.67	Doctorate	411	12.74
Educational services	160	4.96			
Wholesale trade	19	0.59			
Health care or social assistance	592	18.35			
Retail trade	72	2.23			
Arts, entertainment or recreation	43	1.33			
Transportation or warehousing	58	1.80			
Accommodation or food services	31	0.96			
Information	41	1.27			
Other services (except public administration)	38	1.18			
Finance or insurance	190	5.89			
First Responder (including police, fire, ambulance and paramedic)	254	7.87			
Military (including Army, Navy, Air Force and Marines)	475	14.72			
Other	214	6.63			

Annual gross household income was generally high, with 4.1% of participants recording an income less than AUD$40,000 (GBP£23,000/USD$30,000); 14.7% recording an income between AUD$40,000–$75,000 (£23,000–£43,000/$30,000–$58,000); 18.3% recording an income between AUD$75,000–$110,000 (£43,000 and £63,000/$58,000–$85,000); 17.7% recording an income between AUD$110,000–$150,000 (£63,000 and £86,000/$85,000–$115,000); 26.8% recording an income between AUD$150,000–$280,000 (£86,000 and £160,000/$115,000–$215,000); and 12.8% recording an income greater than AUD$280,000 (£160,000/$215,000). A number of participants (5.5%) preferred not to disclose income information.

### Descriptive statistics

The descriptive statistics of the raw scores for each of the constructs measured are provided in Table 2. Because of the skewness of the Exercise score, this variable was log transformed, which rendered it closer to a normal distribution (M = 1.85, SD = 0.36, Min = 0.30, Max = 2.86, Skewness = -0.70, Kurtosis = 1.39). Subsequently, all variables were standardised prior to further analysis.

**Table 2:** Descriptive statistics for each of the variables measured

	N	Mean	SD	Min	Max	Skewness	Kurtosis
Dispositional Resilience score	3336	3.28	0.79	1	5	− 0.30	− 0.12
Dispositional Vulnerability score	3336	1.81	0.69	1	5	1.14	1.31
Social Support Friends score	3335	5.50	1.36	1	7	− 1.14	1.04
Social Support Significant Other score	3335	5.76	1.49	1	7	− 1.49	1.62
Social Support Family score	3335	5.29	1.54	1	7	− 0.99	0.31
Sleep Quality score	3336	2.82	0.72	1	4	− 0.34	0.07
Sleep Amount score	3332	6.98	1.06	4	13	− 0.31	0.71
Exercise score	2581	93.44	70.61	0	720	2.41	10.93
Nutrition score	3334	1.91	0.33	1	3	0.03	− 0.11
Mindfulness score	3336	4.08	1.05	1	6	− 0.20	− 0.50
Gratitude score	3335	6.03	1.06	1	7	− 1.55	2.84
Cognitive flexibility score	3335	5.67	0.98	1	7	− 0.96	1.39
Locus of Control score	3334	3.57	0.63	1	5	− 0.28	0.30
Occupational self-efficacy score	3336	4.80	0.87	1	6	− 0.77	0.64
Job Autonomy score	3323	5.62	1.24	1	7	− 1.34	2.03
Workload score	3335	3.91	1.02	1	5	− 0.65	− 0.47
Work Purpose score	3325	6.51	2.26	0	9	− 1.03	0.46

### Examining the proposed model structure

Support for the proposed Resilience Shield model was tested using Structural Equation Modelling (SEM) using diagonally weighted least squares, which is appropriate for ordinal data [56]. Given the large sample size, the Comparative Fit Index (CFI) and Root Mean Square Error of Approximation (RMSEA) are reported to evaluate model fit. CFI values > 0.90 and RMSEA values < 0.08 are considered acceptable fit, while CFI values > 0.95 and RMSEA values < 0.05 are considered good fit [41].

First, the fit of the measurement model for each of the Shield Layers was evaluated. Latent variables were created for each of the four constructs assessed in the Mind Layer: (i) Mindfulness (2 items), (ii) Gratitude (2 items), (iii) Cognitive flexibility (2 items), and (iv) Locus of Control (2 internal items and 2 reverse-coded external items). A Mind latent construct was created from the latent variables Mindfulness, Gratitude, Cognitive flexibility, and Locus of Control. This model showed a good fit, χ2(31) = 242.39, *p* < 0.001, CFI = 0.96, RMSEA = 0.05.

Similarly, a Body latent variable was created from the manifest variables Sleep Quality score, Sleep Amount score, Exercise score, and Nutrition score. This model showed a poor fit, χ2(2) = 140.80, *p* < 0.001, CFI = 0.83, RMSEA = 0.17. Parameter estimates showed that the Exercise score (R^2^ = 0.029) and Nutrition score (R^2^ = 0.057) contributed very little to the model, relative to the Sleep quality score (R^2^ = 0.663) and the Sleep amount score (R^2^ = 0.424).

Next, a latent variable was created for each of the three constructs assessed in the Social Layer: (i) Social support from friends (2 items), (ii) Social support from a significant other (2 items), and (iii) Social support from family (2 items). Subsequently, a Social latent variable was created from the latent variables Social support from friends, Social support from a significant other, and Social support from family. This model showed an acceptable fit, χ2(6) = 7.10, *p* = 0.312, CFI = 1.00, RMSEA = 0.008.

Finally, latent variables were created for each of the four constructs assessed in the Professional Layer: (i) Occupational self-efficacy (2 items), (ii) Job autonomy (2 items), (iii) Workload (2 items), and (iv) Work Purpose (2 items). A Professional latent construct was created from the latent variables Occupational self-efficacy, Job autonomy, Workload, and Work Purpose. This model showed a good fit, χ2(16) = 95.18, *p* < 0.001, CFI = 0.99, RMSEA = 0.04.

To represent resilience as a function of the four Shield Layers, a Resilience latent construct was created from the latent variables Mind, Body, Social, and Professional.

The results of the SEM analysis are reported in Table 3, where numbers between columns represent standardised estimates of the relationships between variables, and numbers between brackets represent the standardised error variance for each variable. These results show a good model fit for the Resilience Shield model, χ2(335) = 2092.60, *p* < 0.001, CFI = 0.94, RMSEA = 0.05. The estimates for the Mind layer latent construct however indicate the presence of a Heywood case, as the standardized estimate of the relationship with the Resilience latent factor is larger than one, and the error variance is negative. This Heywood case is unlikely to be caused by an identification problem, as the measurement model for the Mind Layer was acceptable. It is possible for standardized values over 1.0 to be valid in a correlated model usually when there is strong multicollinearity between two variables [31, 45], e.g. if the Mind Layer is strongly correlated with resilience.

**Table 3:** Standardised estimates (Std. est.) of the relationships between variables (in the columns between variables) and error variance for each variable (in brackets).

Resilience latent variable	Std. est.	Layer latent variable	Std. est.	Subconstruct latent variable	Std. est.	Manifest variable	
Resilience	1.084	Mind	0.526	Mindfulness	0.825	Item 1	(0.319)
(1)		(− 0.176)		(0.724)	0.731	Item 2	(0.466)
			0.691	Gratitude	0.806	Item 1	(0.351)
				(0.523)	0.779	Item 2	(0.393)
			0.422	Cognitive flexibility	0.687	Item 1	(0.527)
				(0.822)	0.838	Item 2	(0.297)
			0.429	Locus of Control	0.408	Item 1	(0.834)
				(0.816)	0.274	Item 2	(0.925)
					0.586	Item 3	(0.656)
					0.701	Item 4	(0.509)
	0.43	Body	0.71			Sleep Quality	(0.496)
		(0.815)	0.481			Sleep Amount	(0.769)
			0.206			Exercise	(0.958)
			0.431			Nutrition	(0.814)
	0.561	Social	0.639	Social support significant other	0.906	Item 1	(0.18)
		(0.686)		(0.592)	0.935	Item 2	(0.125)
			0.751	Social support friend	0.911	Item 1	(0.171)
				(0.436)	0.861	Item 2	(0.259)
			0.822	Social support family	0.874	Item 1	(0.235)
				(0.324)	0.874	Item 2	(0.236)
	0.77	Professional	0.617	Occupational self-efficacy	0.854	Item 1	(0.271)
		(0.407)		(0.619)	0.85	Item 2	(0.278)
			0.671	Job Autonomy	0.851	Item 1	(0.276)
				(0.549)	0.884	Item 2	(0.218)
			0.235	Workload	0.872	Item 1	(0.24)
				(0.945)	0.85	Item 2	(0.278)
			0.7	Work Purpose	0.908	Item 1	(0.175)
				(0.51)	0.945	Item 2	(0.107)

### Contributions to dispositional vulnerability and resilience scores

To examine whether each of the constructs measured makes a unique contribution to resilience, two regression analyses were conducted. After controlling for age and gender (coded as binary with − 1 female and 1 male), all variable scores were entered simultaneously, with Dispositional Resilience scores as the outcome variable in the first model, and Dispositional Vulnerability scores as the outcome variable in the second model.

The results are presented in Table 4. The first model predicting Dispositional Resilience scores was significant, F(17,2451) = 85.14, *p* < 0.001, with 37.1% of the variance explained. Being female was associated with higher resilience. All other variables were significant predictors of Dispositional Resilience scores, with the exception of the Social Support Friends score, the Job Autonomy score, and the Workload score. Most significant relationships were in the expected direction, with higher dispositional resilience being related to more social support from family, more mindfulness, gratitude, and cognitive flexibility, a more internal locus of control, higher occupational self-efficacy and sense of purpose at work, better sleep quality and nutrition, and more exercise. Two counter-intuitive effects are noted, with less support from a significant other and less sleep being associated with higher resilience after accounting for all other variables. The standardised estimates show that largest unique contributions come from constructs related to the Professional layer, in particular occupational self-efficacy and a sense of purpose at work.

**Table 4:** Standardised estimates (Std. est.) of the unique relationships between predictor variables and dispositional resilience and vulnerability scores.

	Dispositional Resilience			Dispositional Vulnerability		
	SE	t	*p*	SE	t	*p*
H0 Age	− 0.03	− 1.43	0.153	− 0.06	− 2.81	0.005
H0 Gender	− 0.12	− 6.15	< .001	− 0.04	− 2.06	0.039
Mindfulness	0.10	5.69	< .001	− 0.20	− 10.46	< .001
Gratitude	0.12	6.12	< .001	− 0.21	− 10.23	< .001
Cognitive flexibility	0.10	5.34	< .001	− 0.03	− 1.42	0.157
Locus of Control	0.07	4.06	< .001	− 0.08	− 4.34	< .001
Sleep Quality	0.11	5.34	< .001	− 0.08	− 3.68	< .001
Sleep Amount	− 0.06	− 3.01	0.003	0.00	− 0.14	0.892
Exercise	0.05	2.94	0.003	− 0.01	− 0.34	0.735
Nutrition	0.10	5.78	< .001	0.01	0.70	0.486
Social Support Friends	0.01	0.72	0.474	− 0.10	− 4.73	< .001
Social Support Significant Other	− 0.06	− 2.91	0.004	0.03	1.24	0.215
Social Support Family	0.05	2.36	0.018	− 0.06	− 2.45	0.014
Occupational self-efficacy	0.30	15.85	< .001	− 0.05	− 2.62	0.009
Job Autonomy	− 0.04	− 1.85	0.065	− 0.03	− 1.23	0.218
Workload	0.02	0.97	0.333	0.06	3.26	0.001
Work Purpose	0.15	7.35	< .001	− 0.11	− 5.31	< .001

H0 indicates estimates as included in the null model

The second model predicting Dispositional Vulnerability scores was also significant, F(17,2451) = 56.83, *p* < 0.001, with 28.3% of the variance explained. Interestingly, being younger and female was also associated with higher dispositional vulnerability. All other variables were significant predictors of Dispositional Vulnerability scores, with the exception of the Social Support Significant Other score, the Cognitive Flexibility score, the Job Autonomy score, the Sleep amount, the Exercise score, and the Nutrition score. All significant relationships were in the expected direction, with higher vulnerability being related to less social support from friends and family, less mindfulness and gratitude, a more external locus of control, lower occupational self-efficacy and sense of purpose at work, higher workload, and poorer sleep quality. The standardised estimates show that largest unique contributions come from constructs related to the Mind layer, in particular gratitude and mindfulness.

## Discussion

The aim of the current study was to evaluate the empirical support for The Resilience Shield model, a recently developed theoretical model of the mechanisms underlying individual resilience, notable for its multi-dimensional approach [80]. The model was evaluated firstly by examining whether each of the constructs identified in each of the Layers contributed a “Layer” latent construct, and whether each of the layers contributed to a higher order latent construct (“resilience”). Secondly, the relevance of each of the mechanisms included in the model was evaluated by examining whether each of these constructs explained unique variance in observed resilience scores.

When examining each of the Shield Layers individually, the results showed acceptable fit of the measurement model for the Social, Mind, and Professional Layers, but poor fit for the Body Layer. These results support the idea that there is a shared factor contributing to scores on social support from friends, family, and a significant other. This is consistent with theory proposing that these factors are related [78, 109], and previous research showing medium to large correlations between these constructs [30].

Similarly, results support the presence of a latent factor contributing to scores on mindfulness, gratitude, cognitive flexibility, and locus of control. This is broadly consistent with previous research showing relationships between some of these constructs, although no previous research has examined the relationships between all four of these constructs. For example, Sawyer et al. [84] found evidence of an indirect effect between mindfulness, perspective taking (the cognitive capacity to consider the viewpoint of others, similar to the cognitive flexibility construct included in The Resilience Shield model) and gratitude. Locus of control has also been shown to be associated with gratitude and mindfulness, with individuals with a more internal locus of control having a greater disposition for gratitude and mindfulness [100]. Watkins et al. [100]’s explanation for the association with gratitude was that individuals with a strong internal locus of control may not expect benefits from others, and thus they experience more gratitude when these unexpected benefits do arise.

Lastly, the measurement model of the Professional Layer suggests there is a shared factor contributing to scores on occupational self-efficacy, job autonomy, workload, and work purpose. This was also an expected finding, as the interrelationships between these constructs have been well documented in the work psychology and leadership literature (e.g. [19, 33, 68]).

In contrast to the other layers, the measurement model of the Body Layer displayed poor fit. One possible explanation for this is that there is no common factor contributing to scores on sleep, exercise, and nutrition. However, this explanation seems unlikely, given the known intercorrelations between these constructs (e.g. [1, 5, 107]). Another possible explanation is that the assessment tools used to index the constructs in this layer are sub-optimal. Indeed, while other constructs in the model were indexed by previously validated instruments, the same was not true for the assessment nutrition, which was done through a custom developed instrument. An alternative well-validated measure is the short Food Frequency Questionnaire, although this instrument still consists of 39 items and takes over 10 min to complete [49]. In addition, the assessment of physical activity could be improved by including a measure of sedentary behaviour as the WHO has included the reduction of sedentary behaviour as a key recommendation in their 2020 guidelines [13].

The SEM results further showed good model fit for a model where each of the four latent layer constructs loaded onto a higher order latent construct. This suggest there is a general factor these layers have in common, as proposed by The Resilience Shield model. As such, consistent with this model this communality between the layers was interpreted as resilience (although of course other interpretations are possible, such as general wellbeing or quality of life). The standardised estimates suggest that the Mind layer makes the biggest contribution to the latent resilience factors, followed by the Professional layer, Social layer, and Body layer. There is currently little literature that has conducted direct comparisons of the contribution of different dimensions to individuals’ resilience, however these results are broadly consistent with research showing that intrapersonal factors are more strongly related to mental health as compared to interpersonal and behaviour factors (e.g. [7, 40]). These data thus provide general support for multidimensional models of resilience, and provide a framework to further investigate the relative contribution of each dimension.

The regression analyses showed that each of the constructs included in The Resilience Shield survey explain independent variance in either dispositional resilience scores, or dispositional vulnerability scores. The only exception to this was job autonomy, which only showed a marginally significant association with dispositional resilience. Moreover, even though the zero-order correlation between job autonomy and dispositional resilience was positive (r(3336) = 0.242, *p* < 0.001), once the other predictors were included in the model this association changed to a negative relationship. This may be due to the medium and large sized correlation between job autonomy and occupational self-efficacy (r(3336) = 0.368, *p* < 0.001) and work purpose (r(3336) = 0.502, *p* < 0.001), respectively, which may have resulted in the residual variance in job autonomy not contributing meaningfully to individual differences in resilience. The same issue may explain the negative contribution of social support from a significant other to dispositional resilience, as this variable is highly correlated with the two other social support variables (both r > 0.4).

These results provide compelling evidence that constructs across a range of dimensions make additive contributions to the explanation of resilience. It thus appears there is validity in considering the combined influence of these dimensions, at least in populations represented by the current sample [92]. Given the increased interested in such multidimensional approaches, the current study makes a considerable contribution to existing literature by illuminating the independence of these contributions. Interestingly, while about half of the variables included significantly predict both dispositional resilience and dispositional vulnerability (in opposite directions), some variables only predict one of these constructs. In particular, social support from friends and workload predicted only dispositional vulnerability, whereas cognitive flexibility, sleep, exercise, and nutrition only predicted dispositional resilience. This shows the value of considering both of these dimensions of resilience in future research.

While one of the strengths of The Resilience Shield Model is its multidimensional approach, there are other dimensions not currently included in the model that are also known to contribute to resilience. Iacoviello and Charney [43] describe a range of psychosocial factors contributing to resilience after trauma exposure. They subdivide these factors into a cognitive component (akin to the Mind layer of The Resilience Shield model), behaviour component (incorporating constructs described in the Body layer and Social layer), and an existential component. The latter component includes some constructs that are included in the Innate layer of The Resilience Shield model, such as a personal moral compass (i.e. values), but also constructs that go beyond the self and instead focus more on environmental-contextual processes. One such example is faith or spirituality which has been shown to positively influence psychological adjustment to stress [3]. Other existential factors relate to social capital, such as social embeddedness and place attachment [43, 71]. Expanding the Resilience Shield to include such environmental-contextual processes may enhance its capacity to inform individuals’ resilience.

In addition to the poor fit of the measurement model of the Body layer, it is important to acknowledge other limitations of the current study and model. The model was only tested and supported for people currently in the workforce, as the relevance of constructs in the Professional Layer in its present form is limited for those who are not employed. However, these constructs could be relevant to other areas of life. Many people who are not in the workforce have other responsibilities requiring skill, commitment, and considerable time investment (e.g. carers, volunteers, stay-at-home parents). People can vary in the degree to which they feel effective at undertaking these responsibilities (e.g. [28]), in the autonomy they have in these roles (e.g. [61]), in the sense of purpose it brings them (e.g. [53]), and in the workload they experience (e.g. [66]). As such, with only minor changes in phrasing, the Professional layer could be made relevant to a larger proportion of the population.

Another limitation of the current study is that it features a predominantly Australian and overall highly educated sample, therefor results cannot be generalised beyond this sample. There are known differences in the predictors of resilience between cultures. For example, in individualistic cultures, personal belief in a just world (the belief that you as a person are fairly treated) is more important to resilience than general belief in a just world (the belief that people generally are fairy treated), which is more important to resilience in collectivist cultures [105]. Moreover, culture and socio-cultural context can influence the development of resilience [21, 22], therefore taking this context into account could be important to understanding resilience in culturally diverse populations. Further research is needed to replicate these findings in more culturally diverse samples, as well as to identify other factors that may additionally contribute to the prediction of individual differences in resilience in such samples.

## Conclusion

Overall, these results suggest that, in the current sample, The Resilience Shield is an acceptable model to index individuals’ performance on a range of indicators that contribute to resilience. As such, the model and its operationalisation through the survey as described above can provide a useful platform to describe individuals’ resilience, and to form the basis of the evaluation of resilience interventions. At present, the instruments available to measure resilience directly are lacking in either reliability or validity [104] hence this multi-dimensional approach may be an apt alternative. In the assessment of resilience, this approach can show individuals which specific areas may be lacking and where efforts can be made to improve resilience (something which is not possible with general scales such as the Brief Resilience Scale). In the evaluation of interventions, a multidimensional approach is superior in its capacity to identify which mechanisms have been targeted in the intervention and which remain unchanged, thereby offering more information about how future interventions can be improved [23, 57].

## Data Availability

The data that support the findings of this study are available from The Resilience Shield but restrictions apply to the availability of these data, which were used under license for the current study, and so are not publicly available. Data are however available from the authors upon reasonable request and with permission of The Resilience Shield.

## Abbreviations

CD-RISC: Connor-Davison Resilience Scale
SEM: Structural Equation Modelling
CFI: Comparative Fit Index
RMSEA: Root Mean Square Error of Approximation
